# Comparative transcriptome profiling of resistant and susceptible rice genotypes in response to the seedborne pathogen *Fusarium fujikuroi*

**DOI:** 10.1186/s12864-016-2925-6

**Published:** 2016-08-11

**Authors:** Slavica Matić, Paolo Bagnaresi, Chiara Biselli, Luigi Orru’, Greice Amaral Carneiro, Ilenia Siciliano, Giampiero Valé, Maria Lodovica Gullino, Davide Spadaro

**Affiliations:** 1 AGROINNOVA – Centre of Competence for the Innovation in the Agro-environmental Sector, University of Turin, Largo Paolo Braccini 2 (ex-Via L. da Vinci 44), 10095 Grugliasco, TO Italy; 2 Department of Agricultural, Forestry and Food Sciences (DISAFA), University of Turin, Largo Paolo Braccini 2 (ex-Via L. da Vinci 44), 10095 Grugliasco, TO Italy; 3 Council for agricultural research and economics (CREA), Genomics Research Centre, via S. Protaso, 302 I −29017, Fiorenzuola d’Arda, PC Italy; 4 Council for agricultural research and economics (CREA), Rice Research Unit, S.S.11 to Torino, km 2,5, 13100 Vercelli, Italy

**Keywords:** Bakanae disease, *Fusarium fujikuroi*, Gibberellin, Plant-fungus interaction, Rice, RNA-seq, Transcriptome

## Abstract

**Background:**

*Fusarium fujikuroi* is the causal agent of bakanae, the most significant seed-borne disease of rice. Molecular mechanisms regulating defence responses of rice towards this fungus are not yet fully known. To identify transcriptional mechanisms underpinning rice resistance, a RNA-seq comparative transcriptome profiling was conducted on infected seedlings of selected rice genotypes at one and three weeks post germination (wpg).

**Results:**

Twelve rice genotypes were screened against bakanae disease leading to the identification of Selenio and Dorella as the most resistant and susceptible cultivars, respectively. Transcriptional changes were more appreciable at 3 wpg, suggesting that this infection stage is essential to study the resistance mechanisms: 3,119 DEGs were found in Selenio and 5,095 in Dorella. PR1, germin-like proteins, glycoside hydrolases, MAP kinases, and WRKY transcriptional factors were up-regulated in the resistant genotype upon infection with *F. fujikuroi*. Up-regulation of chitinases and down-regulation of MAP kinases and WRKY transcriptional factors were observed in the susceptible genotype. Gene ontology (GO) enrichment analyses detected in Selenio GO terms specific to response to *F. fujikuroi*: ‘response to chitin’, ‘jasmonic acid biosynthetic process’, and ‘plant-type hypersensitive response’, while Dorella activated different mechanisms, such as ‘response to salicylic acid stimulus’ and ‘gibberellin metabolic process’, which was in agreement with the production of gibberellin A_3_ in Dorella plants.

**Conclusions:**

RNA-seq profiling was performed for the first time to analyse response of rice to *F. fujikuroi* infection. Our findings allowed the identification of genes activated in one- and three- week-old rice seedlings of two genotypes infected with *F. fujikuroi*. Furthermore, we found the pathways involved in bakanae resistance, such as response to chitin, JA-dependent signalling and hypersensitive response. Collectively, this provides important information to elucidate the molecular and cellular processes occurring in rice during *F. fujikuroi* infection and to develop bakanae resistant rice germplasm.

**Electronic supplementary material:**

The online version of this article (doi:10.1186/s12864-016-2925-6) contains supplementary material, which is available to authorized users.

## Background

Bakanae disease or disease of foolish seedlings is one of the most important seed-borne diseases of rice, caused by the fungal pathogen *Fusarium fujikuroi* Nirenberg [teleomorph *Gibberella fujikuroi* (Sawada) Ito in Ito & K. Kimura] [1, 2]. Common disease symptoms include elongated seedlings which may also be stunted and yellow with crown rot [3, 4]. As many *Fusarium* species, *F. fujikuroi* is a necrotrophic pathogen [5].

Crop losses due to bakanae disease may reach 40 % in an outbreak or epidemic [6]. Reduced pesticide availability for seed dressing in recent years led to increased bakanae disease incidence in several countries, becoming a serious threat for rice cultivation [7]. Though many rice cultivars (cvs.) have been screened for resistance to bakanae disease [8–10], to date no cultivar has shown a complete resistance, and despite the advancements in elucidating the *F. fujikuroi* genome [11, 12], there is still a limited knowledge on the mechanisms of rice resistance to bakanae, crucial for the development of appropriate control strategies.

Response of rice to fungal attack is complex and involves a battery of biological and physiological processes. RNA-seq provides enhanced detection potential over more traditional approaches as microarrays with respect to detection of previously unknown transcripts, production of low background signal, wide dynamic range of expression levels, and ability to detect sequence modifications such as SNPs in the transcribed regions [13]. Recently, RNA-Seq was employed in characterizing transcriptional resistance mechanisms to rice pathogens (e.g. [14–16]), but to the best of our knowledge, the response of rice to *F. fujikuroi* still awaits investigation by RNA-Seq.

In this study, the transcriptional response of two rice cultivars to *F. fujikuroi* infection was analysed at two time points of inoculation by using RNA-Seq. The two cultivars, Selenio and Dorella, were selected after dedicated screenings as the most resistant and susceptible to bakanae disease, respectively. Our study identified novel genes involved in rice resistance to bakanae disease and may contribute in a deeper understanding of the rice-*F. fujikuroi* interaction.

## Results and discussion

### Evaluation of rice cultivars against bakanae disease

**Fig. 1:**
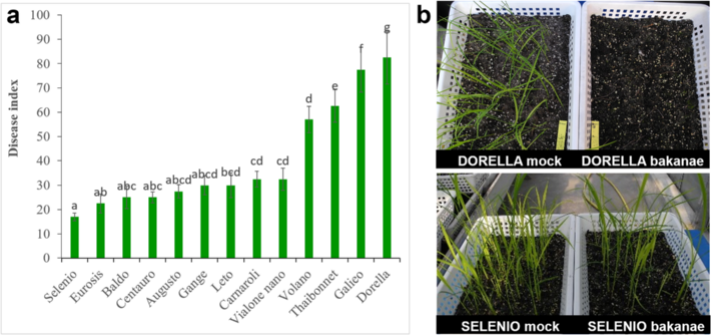
Rice cultivars screened for resistance to *Fusarium fujikroi.* **a** disease index and (**b**) symptomatology. Rice mock- and bakanae-inoculated plants were grown in controlled greenhouse conditions. Disease index and disease symptoms were evaluated 3 weeks post germination

Out of 12 genotypes screened against bakanae disease, Selenio resulted the most resistant (disease index 17.0 %), while Dorella turned out to be the most susceptible (disease index 82.5 %). A sporadic presence of yellowish plants, without typical symptoms of bakanae disease was observed in cv. Selenio. On the contrary, typical bakanae-diseased symptoms with elongated and thin internodes which progressively led to plant death were observed in plants of cv. Dorella (69 %). The other tested genotypes showed intermediate levels of resistance (Fig. 1).

**Fig. 2:**
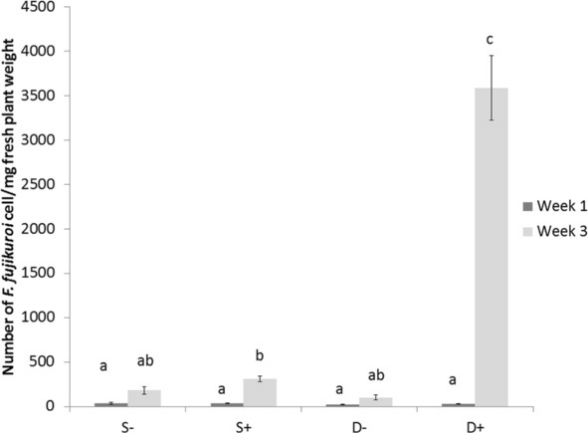
Quantification of *F. fujikuroi* in rice cultivars Selenio and Dorella at 1 and 3 weeks post germination. S- = non inoculated Selenio; S + = bakanae inoculated Selenio; D- = non inoculated Dorella; D + = bakanae inoculated Dorella. Error bars show standard deviations for triplicate assays. Columns with the same letter are not statistically different by Duncan’s Multiple Range Test (*P* < 0.05)

The *F. fujikuroi* colonization was quantified in the Selenio and Dorella genotypes at 1 and 3 weeks post germination (wpg) (Fig. 2). Dorella showed abundant infection with *F. fujikuroi* at 3 wpg (15 times more than Selenio), confirming its sensitivity, while at 1 wpg a low *F. fujikuroi* infection level with no significant differences between the two cultivars was shown. Phenotypic and quantitative molecular differences in Selenio and Dorella inoculated with *F. fujikuroi* allowed to select the two cultivars for further transcriptomic studies.

### Differentially expressed genes in bakanae-infected rice

RNA was isolated from leaves of both cvs. Selenio and Dorella, inoculated with *F. fujikuroi* or with the mock, at 1 and 3 wpg, and subjected to whole transcriptome sequencing via RNA-seq. Selenio was constituted in 1987, while Dorella was constituted in 1998, both in Italy. Both cultivars showed similar growth rate and developmental stage during the sampling period. The growing cycle from sowing to spike lasts 95 days for both cultivars and the growing cycle from sowing to maturity lasts, respectively, 145 days for Selenio (plant height: 76.0 cm; ear length: 17.0 cm) and 140 days for Dorella (plant height: 96.0 cm; ear length: 21.0 cm). Both cultivars are not pigmented and the grains are not aromatic and not glutinous. Three biological replicates were sequenced for each genotype (Selenio *vs* Dorella), disease condition (mock *vs* bakanae-inoculated), and infection time point (1 *vs* 3 wpg). Raw reads generated from the Illumina Genome Analyzer GAIIx were filtered by Illumina passed-filter call. Subsequently, adapters identified by fastQC and low-quality regions were filtered out by cutadapt application [17]. Filtered, 51-base reads for each biological replicate (on average, 17 million reads, Additional file 1: Table S1) were mapped with Bowtie/2TopHat2 to the rice genome sequence (*Oryza sativa* Japonica - Nipponbare IRGSP-1.0.20).

Read counts were generated from Bam alignment files with HTSeq software [18]. Data normalization and call of differentially expressed genes (DEGs) was implemented with DESeq2 R package [19, 20].

Pearson correlation coefficients for normalized expression values of samples are shown in Additional file 2: Table S2. All biological replicates sharing cultivar type (Selenio *vs* Dorella), infection time point (1 *vs* 3 wpg) and treatment (mock *vs* bakanae-inoculated) showed correlation coefficients above 0.9 indicating a good reproducibility between biological replicates. Expression values of all detected transcribed genes and non-coding RNAs are reported as DESeq-normalized read counts and log_2_ fold changes in Additional file 3: Table S3.

The number of DEGs at 1 wpg was 80 in Selenio (Selenio mock 1 wpg *vs* Selenio inoculated 1 wpg) of which 24 were Selenio-specific expressed loci, 5 were common DEGs, 7 were genes called as DEGs in Selenio but expressed loci also in Dorella, and 44 were DEGs common in both cultivars at 3 wpg; and 1,285 in Dorella (Dorella mock 1 wpg *vs* Dorella inoculated 1 wpg) of which 797 were Dorella-specific expressed loci, 7 were expressed loci in Dorella but called as DEGs also in Selenio, 5 were common DEGs, and 476 were DEGs common in Selenio and Dorella at 3 wpg.

**Fig. 3:**
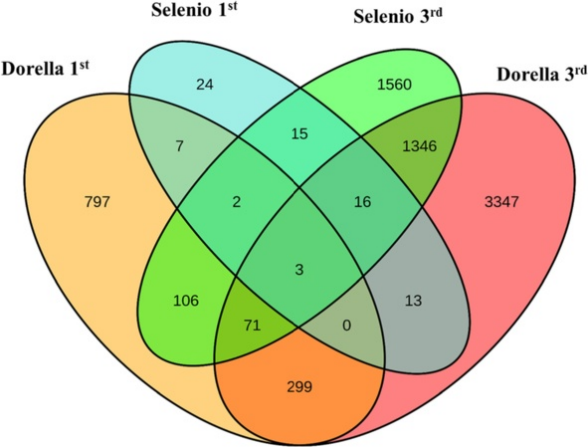
Venn diagrams of DEGs modulated by bakanae disease. Venn diagrams represent DEGs in resistant (Selenio) and susceptible (Dorella) rice genotypes after *F. fujikuroi* inoculation at 1 and 3 weeks post germination

The number of DEGs was higher at 3 wpg in both cultivars: 3,119 in Selenio (Selenio mock 3 wpg *vs* Selenio inoculated 3 wpg), of which 1,560 were Selenio-specific expressed loci, 19 were common DEGs, 1,346 were genes called as DEGs in Selenio but expressed loci also in Dorella, and 194 were DEGs common in Selenio and Dorella at 1 wpg; and 5,095 in Dorella (Dorella mock 3 wpg *vs* Dorella inoculated 3 wpg), of which 3,347 were Dorella-specific expressed loci, 1,346 were expressed loci in Dorella but called as DEGs also in Selenio, 19 were common DEGs, and 383 were DEGs common in both cultivars at 1 wpg (Fig. 3).

Modulated genes for both genotypes were therefore more abundant at 3 wpg than at 1 wpg, indicating a higher infection level in mature rice plants compared to younger plants [21]. Rice plants germinated one week after sowing, and the first bakanae symptoms occurred at 1 wpg. Susceptible cultivars of rice, inoculated with pathogenic *F. fujikuroi,* started to die at 3 wpg, indicating that this is another crucial time point [22]. The higher number of DEGs identified in the susceptible cv. Dorella compared to the resistant cv. Selenio is consistent with a highly visible infection of *F. fujikuroi* in the tissues of Dorella, resulting in a numerically higher number of transcriptionally modulated genes. This finding is similar to what has been described for the interaction between rice and other rice pathogens, where the susceptible cultivar showed much more DEGs than the resistant one [14, 16]. However, the defence mechanisms involved in the bakanae disease responses were largely divergent between Selenio and Dorella since the two genotypes shared only 5 and 19 DEGs at 1 and 3 wpg, respectively.

**Fig. 4:**
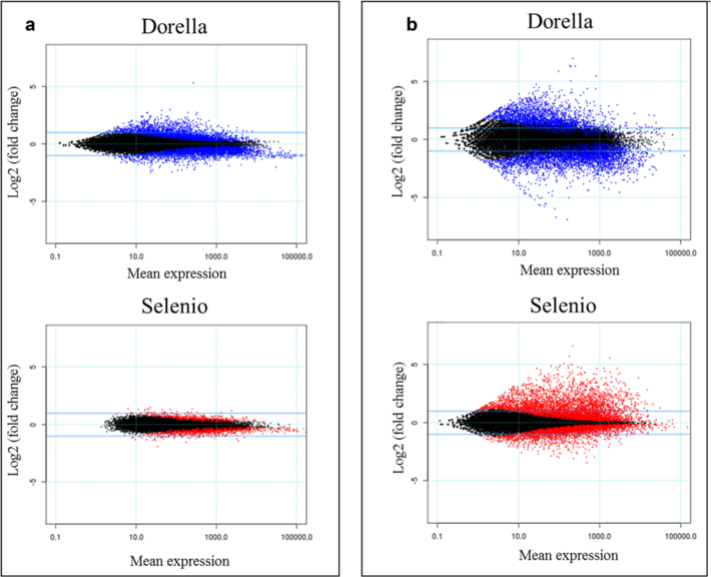
Mean expression versus log fold change plots (MA-plots). Transcriptional changes are presented in Selenio and Dorella at 1 week post germination (**a**) and 3 weeks post germination (**b**). Normalized mean expression values are plotted versus log2 fold changes. Genes with an FDR < 0.05 are plotted in blue for Dorella and red for Selenio

The proportion of up-regulated DEGs was higher in the susceptible genotype (62.0 %) with respect to the resistant one (38.8 %) at 1 wpg, whereas it was higher in the resistant genotype (75.9 %) compared to the susceptible one (41.2 %) at 3 wpg, as indicated in the MA-plots (Fig. 4). Indeed, a higher proportion of down-regulated genes was observed in Dorella at 3 wpg (58.8 %) when systemic infection, characterized by specific symptoms of bakanae in all the green tissues of rice, was well established.

Functional annotations of the rice transcripts available from RAP-DB (http://rapdb.dna.affrc.go.jp/) were used to investigate the functions and the role of the selected genes differentially expressed in response to *F. fujikuroi* infection. The criteria used for the description of genes in the following sections were based either on the higher fold changes (FC) in cv. Selenio in the case of cytochrome P450 monooxygenases, or on the defence role of genes already reported in rice infected with other pathogens [14, 15, 23–26].

### Cytochrome P450 monooxygenases

Cytochrome P450 monooxygenases (P450s) are universal enzymes that catalyse the oxidation of many substrates by the activation of molecular oxygen. Plant P450s catalyse several enzymatic steps for various plant metabolites such as defence-related metabolites, phytohormones, pigments, fatty acids, and lignin [27, 28]. Here, we found a putative cytochrome P450 (Os12g0582700; family 94) as the most up-regulated gene in Selenio (3 wpg) with a log_2_FC 6.60 (Additional file 4: Table S4). Other highly up-regulated P450s in Selenio (3 wpg), also located on chromosome 12, were Os12g0443000 and Os12g0150200 (both genes belonging to family 94). The three P450s were not DEGs either in Selenio or in Dorella at 1 wpg. Interestingly, P450 Os12g0443000 was the highest down-regulated gene in Dorella with log_2_FC -3.66 at 3 wpg, while the other two P450s were not present among the DEGs in this cultivar.

### PR genes

Pathogenesis-related (PR) proteins have antifungal activity against many phytopathogenic fungi, such as *Phytophthora infestans*, *Phytophthora parasitica*, *Uromyces fabae,* and *Erysiphe graminis* [29, 30]. PR1 plant proteins have homology to the superfamily of cysteine-rich proteins, and when act as protease inhibitors they can suppress the enzymatic activities in response to proteinase attack produced by plant pathogens [31, 32]. PR1 genes were induced in wheat infected by *Fusarium pseudograminearum* and in rice infected by *Magnaporthe oryzae* [15, 23, 33].

We found, respectively five and six up-regulated PR1 genes in Selenio and in Dorella at 3 wpg (Additional file 5: Table S5). Furthermore, there were no down-regulated proteinase inhibitors in Selenio, while in Dorella five of them were down-regulated. Interestingly, out of five up-regulated genes in Selenio (all proteinase inhibitors), three of them were down-regulated in Dorella (Os01g0615050, Os03g0429000 and Os01g0127600). At 1 wpg, there were no induced PR1 genes in Selenio, whereas only one PR1 gene was transcriptionally activated in Dorella.

Few other PR genes, including thaumatin-like genes, were identified as modulated by *F. fujikuroi* infection mainly at 3 wpg in Selenio and Dorella (4 and 8 genes, respectively). Out of four up-regulated PR genes in Selenio, three were down-regulated in Dorella (Os01g0693400, Os12g0555300, and Os12g0569500; Additional file 5: Table S5). Furthermore, the four up-regulated genes in Selenio were also induced in rice during *M. oryzae* infection (Os01g0693400 and Os12g0569500 in compatible interactions, and Os04g0398000 and Os12g0555300 in incompatible interactions) [15].

### Glycoside hydrolases

Glycoside hydrolases (GH) catalyse the hydrolysis of glycosidic bonds in cell wall polymers. The rice GH are classified into 34 families encoding 437 GH genes [26]. The largest GH17 family contains PR2 genes producing β-1,3-glucanases that hydrolyse β-1,3-D-glucosidic linkages in β-1,3-glucans [34]. Fungal cell walls usually contain both chitins and β-1,3-glucans, and the contemporary expression of chitinases and β-1,3-glucanases indicate their mutual function in fungal cell wall degradation [35]. Sharma et al. [26] found 128 up-regulated and 82 down-regulated GH genes during response of rice to four rice pathogens, whose 19 up-regulated in response to *M. oryzae*, the causal agent of blast disease. Kawahara et al. [15] reported up-regulation of 23 GH17 genes in early-stage of *M. oryzae* infection.

In the present work, an higher up-regulation of GH genes, comprising β-1,3-glucanases, was observed at 3 wpg; 16 genes in Selenio and 13 genes in Dorella (Additional file 6: Table S6). Five up-regulated GH genes (Os01g0713200, Os01g0860800, Os01g0940800, Os03g0792800, and Os11g0704600) in Selenio were in common with the rice-*M. oryzae* interaction [15].

At 1 wpg, Selenio did not induce any GH gene, while Dorella up-regulated 9 glucanase genes. Dorella shared 4 up-regulated GH genes during both infection stages. A higher activation of glucanase genes in the susceptible genotype compared to the resistant one at 1 wpg could be attributed to the *F. fujikuroi* colonization of Dorella plants and to the consequent activation of the mechanisms of fungal cell-wall degradation.

### Chitinases

Chitinases belong to the PR protein families PR3, 4, 8, and 11, and are involved in the plant defence response against pathogens and pests by hydrolysing the chitin in the cell wall of fungi and in the skeleton of insects [36, 37]. Three up-regulated chitinases were common in Dorella at 1 and 3 wpg, while Selenio did not show activation of chitinases at 1 wpg (Additional file 7: Table S7). Interestingly, among the up-regulated chitinases in Selenio at 3 wpg, Os02g0605900 was down-regulated in Dorella during both infection time points. Os02g0605900 was also up-regulated during incompatible interaction between rice and *M. oryzae* [15]. At 3 wpg, we found 10 up-regulated chitinase genes in the susceptible genotype, whereas Selenio showed only 2 up-regulated chitinases. Higher up-regulation of chitinase genes in Dorella compared to Selenio at 3 wpg might be a consequence of fungal colonization of plant cells and subsequent activation of fungal cell-wall degradation, as in the case of glucanase genes at 1 wpg.

### Peroxidases

Peroxidases are PR9 proteins induced in plant tissues upon pathogen infection and they are known as defence-related proteins. Peroxidases are produced to prevent cellular diffusion of pathogens by massive production of reactive oxygen and nitrogen species, and subsequent creation of an highly toxic environment, or by development/reinforcement of structural barriers [38–40]. In our study, we found 12 up-regulated peroxidase genes in Dorella and 7 in Selenio at 3 wpg (Additional file 8: Table S8). Activation of a higher number of peroxidases was also found at 1 wpg in susceptible genotype (9) as compared to the resistant genotype (2). Selenio did not share activated peroxidases between 1 and 3 wpg, while one induced peroxidase was common in Dorella during both infection time points (Os02g0236600). Lower induction of peroxidases in Selenio could be attributable to a reduced spreading of *F. fujikuroi* in this genotype. Three peroxidases (Os01g0787000, Os03g0285700, and Os07g0677600) up-regulated in Selenio were also induced in rice infected with *M. oryzae* [15].

### Germin-like proteins

Oxalate oxidase-like genes, recently known as germin-like protein (GLP) genes, are included in plant defence mechanisms, where some of them increase their expression upon pathogen infection or insect attack [41–44]. GLP genes are known to contribute to rice resistance. Manosalva et al. [24] demonstrated that when GLP genes of chromosome 8 were suppressed, rice plants were more susceptible to rice blast and sheath blight (caused by *Rhizoctonia solani*).

At 3 wpg, 4 up-regulated GLP genes were identified in Selenio, while 5 up-regulated and 2 down-regulated GLP genes were found in Dorella (Additional file 9: Table S9). Interestingly, 2 up-regulated GLPs (Os08g0189600 and Os11g0537350) in Selenio were down-regulated in Dorella. Out of four up-regulated GLPs in Selenio, two genes (Os08g0189600 and Os08g0189400) are located on chromosome 8, where other GLP genes involved in rice fungal resistance are present [24]. On the other hand, Selenio did not activate GLP genes at 1 wpg, whereas Dorella had all GLPs genes up-regulated at this stage.

### MAP kinases

Mitogen-activated protein kinases (MAPKs) play an important role in many resistance (R)-mediated defence responses to plant pathogens [45]. MAPK cascades are involved in a range of signalling pathways downstream of receptor kinases, such as the biosynthesis of phytoalexins in plants [46]. MAPK cascades are composed of three functionally linked protein kinases: MAPK, phosphorylated and activated by a MAPK kinase (MAPKK), which itself is activated by another protein kinase, a MAPKK kinase (MAPKKK) [47].

A MAPK cascade, including the MAPKK OsMKK4 and two MAPKs, OsMPK3 and OsMPK6, is involved in chitin elicitor-induced biosynthesis of diterpenoid phytoalexins in rice [25]. Two of these genes were up-regulated in Selenio at 3 wpg (OsMKK4 or Os02g0787300 with a log_2_FC 3.27, and OsMPK3 or Os03g0285800 with a log_2_FC 1.64), whereas one of them was the most down-regulated MAP-kinase in Dorella (OsMKK4 with a log_2_FC -2.83) (Additional file 10: Table S10). Furthermore, Selenio had five further MAP kinases activated while the majority of MAP-kinases were down-regulated in Dorella (8 genes in total). No MAP kinases were DEGs either in Selenio or in Dorella at 1 wpg.

### WRKY transcriptional factors

WRKY transcription factors are a superfamily of zinc-finger transcription factors which contain a highly conserved DNA-binding WRKY domain. Most WRKY proteins bind to the conserved W-box, which is present in the promoters of many defence-related genes [48, 49].

At 3 wpg, Selenio showed an induction of 22 WRKY genes with FC ranging from 2 to 15 and a down-regulation for only two WRKY genes (Additional file 11: Table S11). Conversely, at the same time point Dorella up-regulated only 4 WRKY genes (FC from 2 to 4), and down-regulated 15 WRKY genes (Additional file 11: Table S11). Selenio showed no activation of WRKY genes at 1 wpg, while Dorella showed 4 WRKY up-regulated genes with only one of them shared between 1 and 3 wpg. Interestingly, 8 up-regulated WRKY genes in Selenio at 3 wpg were down-regulated in Dorella at the same infection time point. On the contrary, one of the down-regulated genes in Selenio (WRKY29) was highly up-regulated in Dorella.

Comparison of WRKY gene induction identified in the present study with analyses carried out on *M. oryzae*-rice interactions [14, 15], highlighted a common activation of WRKY-62, −76, −19, and −50 genes in resistant rice cultivars.

### Expression of defence-related genes in Selenio and Dorella in response to *F. fujikuroi*

**Fig. 5:**
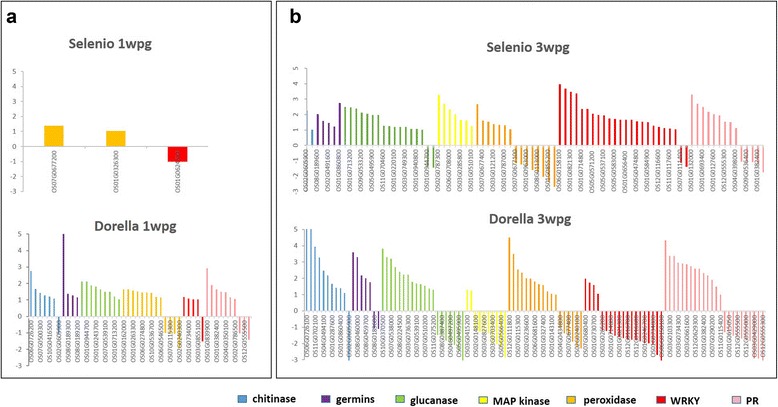
Transcriptome profiling of defence-related genes in Selenio and Dorella in response to *F. fujikuroi*. Two infection time points in two rice genotypes are shown: (**a**) 1 week post germination and (**b**) 3 weeks post germination

Comparison of the transcript profiles for Selenio and Dorella at 1 and 3 wpg reveals a different modulation of the plant response to *F. fujikuroi*. In detail, Selenio activated, albeit to a moderate extent, only peroxidase genes at 1 wpg (Os07g0677200 and Os01g0326300), while many genes related to glucanases, germin-like proteins, peroxidases, MAP kinases, WRKY transcriptional factors, and PR genes were activated at 3 wpg (Fig. 5). Overall, Selenio up-regulated the highest number WRKY transcription factors, MAPK, and glucanases, and down-regulated the majority of peroxidases at 3 wpg. Furthermore, Dorella activated all groups of defence-related genes at both infection time points (with exception of MAP-kinases at 1 wpg), and it activated the highest number of chitinases, glucanases, and peroxidases and down-regulated the majority of WRKY transcription factors at 3 wpg (Fig. 5). Further analyses focused on the results at 3 wpg, because this infection time point with a higher transcriptomic response in both cultivars, showed to be crucial in the *F. fujikuroi*-rice interactions.

### GO enrichment analyses

**Fig. 6:**
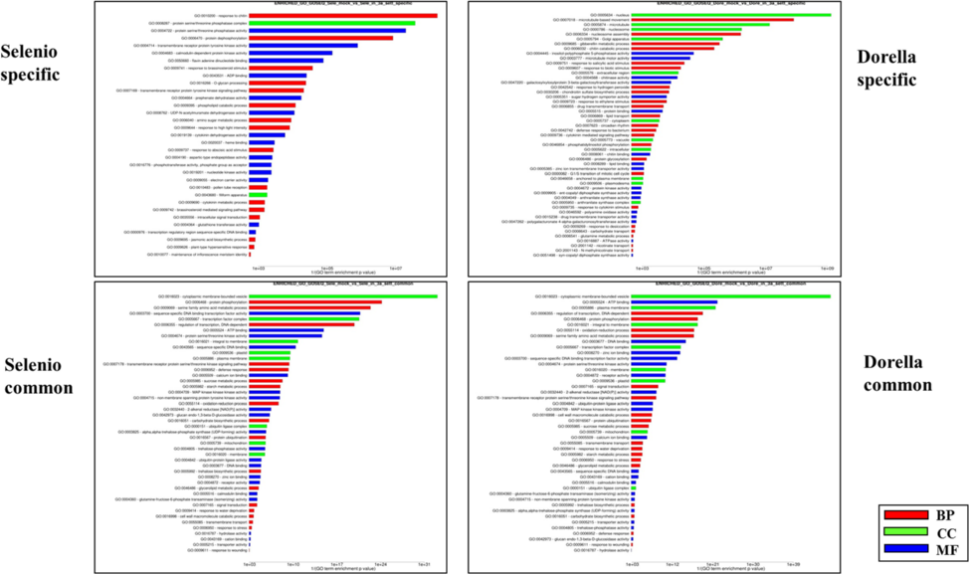
GO enrichment analyses in Selenio and Dorella rice genotypes upon *F. fujikuroi* infection. Common and genotype-specific enriched GO terms were obtained by goseq package using a FDR cutoff of 0.1. GO term enrichment p values are indicated on X axis. Red, blue and green columns refer, respectively, to biological process (BP), molecular function (MF) and cell compartment (CC) GO domains

As GO terms covering genes with longer transcripts are more likely to be determined as enriched due to higher read counts and increased statistical power for DEG call [50], the goseq R package was implemented in order to limit such length-related biases [51]. Test sets for goseq inputs were DEGs assessed by DESeq2 Goseq output, resulting in 79 and 97 enriched GO terms for Selenio and Dorella, respectively. Specific and common GO terms at 3 wpg, i.e. 46 common, 33 Selenio-specific, and 51 Dorella-specific GO terms, are detailed in Fig. 6.

Among 46 common enriched terms in both rice genotypes, some GO terms were associated to general plant response to fungus attack such as ‘defence response’ (GO:0006952), ‘MAP kinase kinase kinase activity’ (GO:0004709), ‘oxidation-reduction process’ (GO:0055114), ‘response to stress’ (GO:0006950), and ‘response to wounding’ (GO:0009611). On the other hand, some GO terms associated with fungal infection were specific.

Among the GO terms found solely in Selenio, but not in Dorella, there are: ‘response to chitin’ (GO:0010200), ‘brassinosteroid mediated signaling pathway (GO:0009742), ‘response to abscisic acid stimulus’ (GO:0009737), ‘jasmonic acid biosynthetic process’ (GO:0009695), ‘plant-type hypersensitive response (GO:0009626), and ‘transmembrane receptor protein tyrosine kinase signalling pathway’ (GO:0007169).

Although Dorella displayed a higher number of specific GO groups, many of them were not associated with a specific plant response to biotic stress. Furthermore, Dorella activated a different battery of response mechanisms to *F. fujikuroi* as compared to Selenio, e.g. ‘response to salicylic acid stimulus’ (GO:0009751), and ‘gibberellin metabolic process’ (GO:0009685).

In the following sections, some Selenio-specific GO terms most likely involved in bakanae resistance (hypersensitive response, jasmonic acid biosynthetic process and response to chitin) and two Dorella-specific GO terms associated with bakanae disease (response to salicylic acid and gibberellin metabolism) are described.

### Hypersensitive response

The interaction of avirulence (Avr) genes in pathogens and disease resistance (R) genes in plants can cause a localized programmed cell death named hypersensitive response (HR) [52]. The protein Avr9 of *Cladosporium fulvum* provokes various defence responses in tomato plants bearing the *Cf9* gene, and after Avr9 recognition the cells close to the infection site die [53, 54]. The GO term ‘plant-type hypersensitive response’ (GO:0009626), enriched only in Selenio (at 3 wpg), includes defence genes encoding Avr9/Cf-9 elicited protein, C2 domain containing protein, *Pto* kinase interactor 1 and chitinase 6, many of them related with HR (Additional file 12: Table S12). On the contrary, all genes comprised in this GO term were down-regulated in Dorella with the exception of putative chitin-binding allergen *Bra r 2*. Thus, activation of a strong hypersensitive response could represent a major differential mechanism conferring resistance to bakanae disease in Selenio.

### Jasmonic acid biosynthetic process

Jasmonic acid (JA), salicylic acid (SA), and ethylene (ET) are phytohormones involved in defence processes. Defence against necrotrophic pathogens and leaf-chewing insects is regulated by ET pathway and JA dependent signalling, while biotrophic pathogens mainly activate SA-dependent signalling pathway [55, 56]. In fact, based on recent studies, gibberellins (GAs) stimulate colonization by necrotrophic fungi, such as *F. fujikuroi,* through suppression of the JA signalling pathway [57].

Genes associated to the GO term ‘jasmonic acid biosynthetic process’ (GO:0009695) were found up-regulated in Selenio (3 wpg) and down-regulated in Dorella (Additional file 13: Table S13). This GO included 12-oxophytodienoic acid reductase, 3-ketoacyl-CoA thiolase, and allene oxide cyclase which participate in the biosynthesis of JA [58–60]. Furthermore, phospholipase D (Os10g0524400 and Os01g0172400) and lipoxygenase-like (Os08g0508800, Os04g0447100, Os08g0509100, and Os03g0700700) genes, necessary for the initial steps of JA synthesis in *Arabidopsis thaliana* [61], were all up-regulated among DEGs in Selenio at 3 wpg. Thus, activation of JA signalling pathway genes may also play a crucial role in the resistance of Selenio to *F. fujikuroi*, while fungal infection and down-regulation of JA-biosynthesis related genes probably induced the suppression of this defence mechanism in Dorella and could be favoured by high production of gibberellins during infection and colonization processes.

### Response to chitin

The GO term ‘response to chitin’ (GO:0010200) was specifically enriched in Selenio; genes associated to this group include various transcription factors, such as heat-stress, GRAS, and ET-responsive genes which were found up-regulated in Selenio, but not in Dorella.

Heat-stress transcription factors are reported to play a role in fungal resistance in other plant species [62, 63]. Selenio up-regulated the heat-stress transcription factors B-2b, B-2c, and A-2a, possibly as a result of a *F. fujikuroi* chitin stimulus (Additional file 14: Table S14). Three transcription factors similar to ET-responsive factors (Os05g0572000, Os05g0420300, and Os03g0860100) were up-regulated in Selenio, but not in Dorella. These factors could indicate that ET-mediated signalling pathway may also be involved in Selenio resistance to *F. fujikuroi*. GRAS transcriptional regulators participate in plant disease resistance and mechanical stress response [64], and the GRAS transcriptional factor Os03g0723000 included in this GO term was found up-regulated in Selenio.

The zinc-finger proteins are known to be involved in rice resistance to pathogens, such as *M. oryzae*, and *Xanthomonas oryzae* pv *oryzae* [65–67]. In this GO group, two zinc-finger RING/FYVE/PHD-type genes (Os03g0240600 and Os01g0755700) were both up-regulated in Selenio and down-regulated in Dorella, and they may contribute to resistance response of Selenio to *F. fujiuroi* (Additional file 14: Table S14).

### Response to salicylic acid stimulus

SA plays an important role in resistance and plant defences against pathogen attacks. R2R3-MYB genes are involved in signalling pathways of salicylic acid [68] and in antagonistic activation of SA defence mechanisms and repression of JA defence mechanisms in *A. thaliana* [69]. In this study, the GO term ‘response to salicylic acid stimulus’ (GO:0009751) was specifically enriched in Dorella. Dorella activated at 3 wpg two P-type R2R3 MYB genes (Os08g0437300 and Os04g0594100), and other putative MYB genes which were either down-regulated in Selenio or not found among DEGs (Additional file 15: Table S15). This indicated that salicylic signalling pathway could be employed in response of Dorella to *F. fujikuroi* attack, but not in Selenio.

### Gibberellin metabolic process

The GA role in stimulating stem growth in rice plants was discovered for the first time during the studies of bakanae disease [70]. The GAs were reported to be produced not only by the causal agent of bakanae disease inducing the stem elongation, reduced root growth, and inhibition of chlorophyll synthesis, but the plant itself is able to synthesize GAs [71]. In our studies, the enzymes involved in the GA synthesis belonging to the GO term ‘gibberellin metabolic process’ (GO:0009685) were up-regulated in Dorella and down-regulated in Selenio at 3 wpg: gibberellin 2-beta-dioxygenase (Os05g0560900), gibberellin 20 oxidase 1 (Os03g0856700), and syn-copalyl diphosphate synthase (Os04g0178300) (Additional file 16: Table S16). Moreover, putative cytochrome P450 dwarf3 gene (Os06g0110000) belonging to this GO term was up-regulated in Dorella, and not included as DEG in Selenio. It was reported that dwarf3 gene encoding a cytochrome P450-type enzyme (or OsKAO gene) is involved in early steps of gibberellin biosynthesis in rice [72]. Interestingly, the dwarf3 gene is involved also in leaf longevity in rice during the stress conditions, and its expression in Dorella is possibly related with pronounced leaf senescence and cell death in response to *F. fujikuroi* beside the gibberellin metabolism [73]*.*

**Fig. 7:**
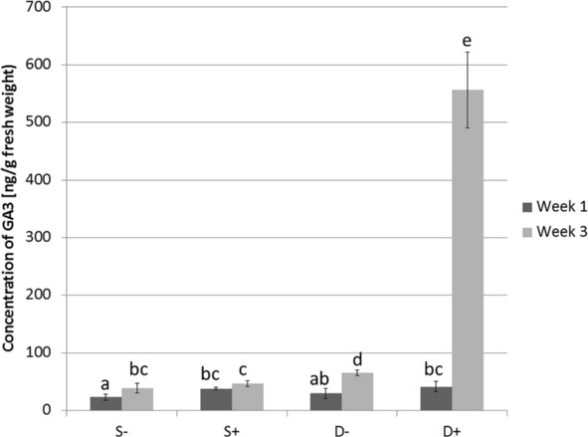
Production of gibberellin A3 by rice cultivars Selenio and Dorella at 3 weeks post germination. S- = non inoculated Selenio; S + = bakanae inoculated Selenio; D- = non inoculated Dorella; D + = bakanae inoculated Dorella. Error bars show standard deviations for triplicate assays. Columns with the same letter are not statistically different by Duncan’s Multiple Range Test (*P* < 0.05)

The activation of gibberellin metabolic genes in the susceptible rice genotype, where typical bakanae symptoms and active growth of pathogen were observed, was in accordance with GA_3_ biosynthesis measured through high performance liquid chromatography (HPLC) analysis. As shown in Fig. 7, Dorella inoculated by *F. fujikuroi* had twelve times more GA_3_ compared to inoculated Selenio at 3 wpg. On the basis of these results, it is evident that the gibberellin group of growth hormones plays important role in response of rice to *F. fujikuroi*. By modulating the production of gibberellins, rice plants are coordinating their level of tolerance to bakanae disease possibly through interaction of the GA-signalling molecules with components of the JA signalling pathway, as already reported under other stress conditions in plants [74], and confirmed in our work by enriched JA metabolism in Selenio.

### KEGG and MapMan maps

**Fig. 8:**
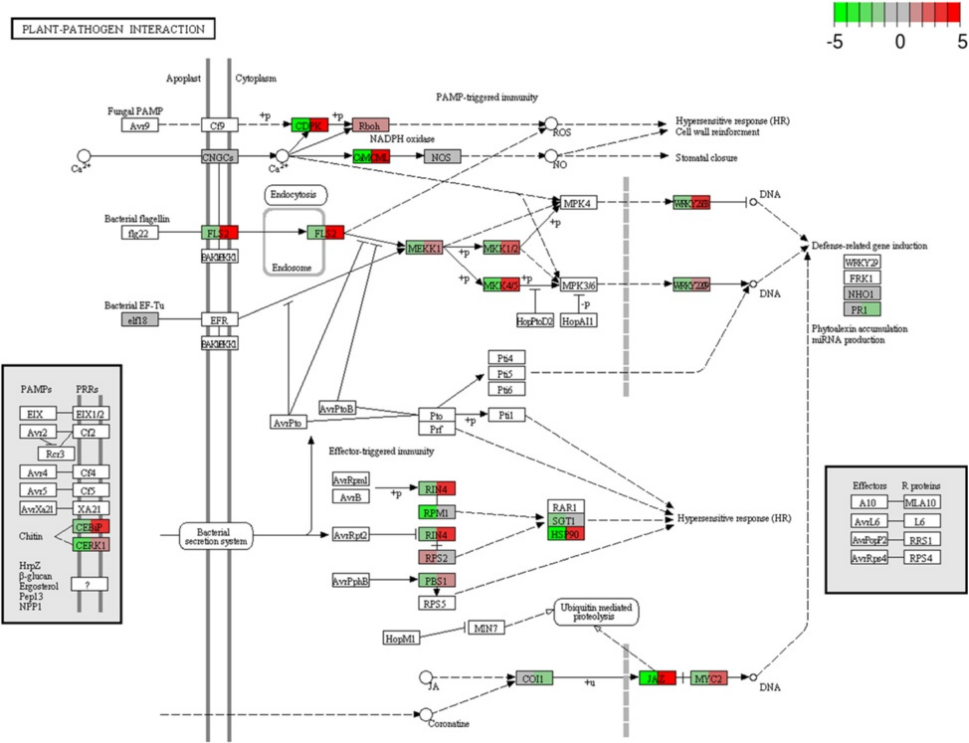
KEGG rice plant-pathogen interaction diagram (osa04626). Coloured boxes are placeholders for one or more genes as assigned to the same KEGG group. For each box, the left side refers to the contrast Dorella mock versus Dorella inoculated (3 wpg) while the right side refers to the contrast Selenio mock versus Selenio inoculated (3 wpg). The colour (in log2 scale) represents the log2-based fold-change (ratio of expression values, inoculated over mock) as indicated in colour key, from -5 (sharp green) to +5 (sharp red)

In order to compare and summarize the response of the two genotypes to infection, the genes were mapped to KEGG rice plant-pathogen interaction diagram (osa04626; http://www.genome.ad.jp/kegg/) [75]. Fold change values for the two contrasts Dorella mock *vs* Dorella inoculated (3 wpg) and Selenio mock *vs* Selenio inoculated (3 wpg) were integrated in the diagram by colour-coding, implementing the pathview R package [76]. This allowed a prompt comparative evaluation of expression responses (Fig. 8). Boxes are placeholders for one or more genes assigned to the same KEGG orthology (KO) group. When more than one gene is mapped to the same group, expression fold-changes were summed up according to default pathview settings.

Based on the plant-pathogen interaction map, when compared to Dorella, Selenio exhibited higher up-regulation of genes in pathways involved in hypersensitive response, response to chitin and JA-dependent signalling confirming the results of the GO enrichment analyses. As an example, following fungal Avr9 and plant Cf9 interaction, Ca^2+^-dependent protein kinase gene (CDPK, e.g. Os01g0808400) involved in hypersensitive response and signal transduction was found up-regulated in Selenio, and down-regulated in Dorella (Fig. 8).

Chitin is a typical microbe-associated molecular pattern (MAMP) molecule from fungal cell walls which elicits plant immune responses. Two plasma membrane proteins, OsCEBiP and OsCERK1 were found necessary to regulate chitin as elicitor of signalling in rice [77, 78]. As summarized in the KEGG map, CEBiP and CERK1 genes were induced in Selenio, but not in Dorella inducing subsequently up-regulation of RIN4 (e.g. Os03g0848600) and HSP90 (e.g.Os06g0716700)-like genes (similar genes are included in these KO groups and fold changes are summed up in the boxes), downstream components of the hypersensitive response (Fig. 8).

Furthermore, JASMONATE ZIM-DOMAIN-like (JAZ; e.g. Os03g0180800) and MYC2 (Os10g0575000), two genes involved in JA signalling, were found by KEGG annotation induced exclusively in Selenio (Fig. 8) which emphasizes the possible role of JA-dependent signalling in Selenio resistance to *F. fujikuroi.*

**Fig. 9:**
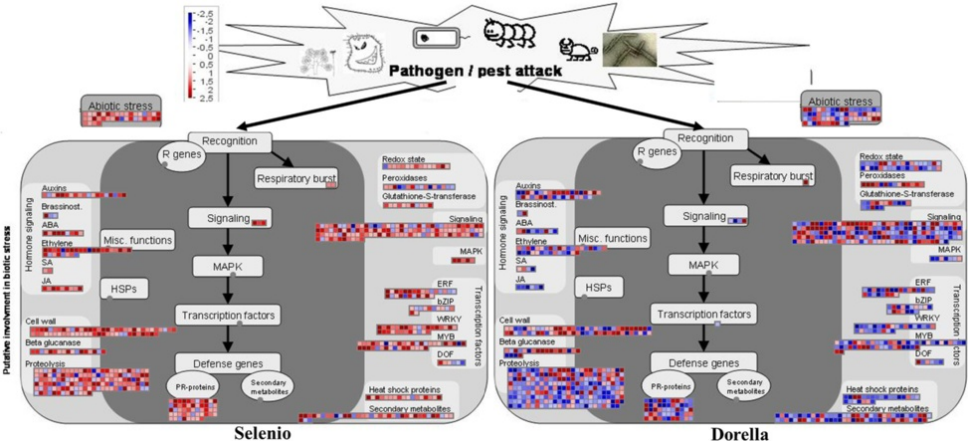
Mapman overview of the genes involved in biotic (*F. fujikuroi*) stress. DEGs of two rice genotypes (Selenio and Dorella) are binned to MapMan functional categories and the values are represented as the log2-transformed values. Red indicates up-regulated genes, whereas blue indicates down-regulated genes. ABA, abscisic acid; bZIP, basic region-leucine zipper; DOF, DNA-binding with one finger; ERF, ethylene response factor; HSP, heat shock protein; JA, jasmonic acid; MAPK, mitogen-activated protein kinase; MYB, myeloblast; PR, pathogenesis-related; R, resistance; SA, salicylic acid

Additionally, employing MapMan software [79], we presented a summary of the main expression changes putatively involved in biotic stress (*F. fujikuroi*) in Selenio and Dorella, as shown by Fig. 9. In contrast to Dorella, Selenio up-regulated the majority of DEGs involved in the candidate resistance pathways already described above, including jasmonic- and ethylene-mediated signalling.

### Validation of RNA-Seq technology

**Fig. 10:**
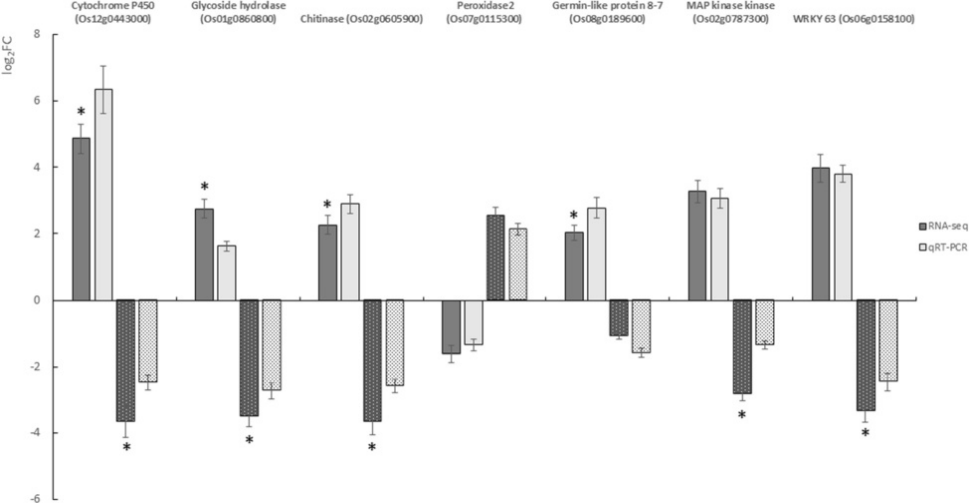
qRT-PCR validation of the relative expression data of genes obtained in RNA-seq analysis. Expression levels of selected transcripts are shown in dark grey (RNA-seq) and light grey (qRT-PCR). Plain columns and dotted columns refer to the expression data in Selenio and Dorella, respectively. EF1α gene was used for transcript normalization of the signal intensity which is shown on the y-axis. The X-axis shows comparisons of the results of two analyses. Error bars show standard deviations for triplicate assays. Asterisks indicate that expression levels are significantly different between RNA-seq and qRT-PCR (unpaired *t* test, *P* < 0.05)

To validate the RNA-Seq technique, seven DEGs were selected based on their expression patterns at 3 wpg for quantitative RT-PCR (qRT-PCR) by using the same RNA extracts as for RNA-seq experiments (Fig. 10). The results of the selected DEGs showed that the qRT-PCR was consistent with the RNA-Seq results showing similar expression pattern of up- and down-regulated genes by using both, RNA-Seq and qRT-PCR, analyses.

## Conclusions

This study presents the first transcriptome from a rice infected by *F. fujikuroi*. We obtained the expression profiles of the resistant rice genotype Selenio and the susceptible genotype Dorella in response to bakanae disease at two infection time points (1 and 3 wpg). More abundant transcriptional changes occurred at 3 wpg in both cultivars, suggesting the importance of this temporal point of infection for studying the rice bakanae resistance mechanisms.

The transcriptomic analysis allowed a remarkable insight into novel genes and pathways involved in defence of rice distinguishing two types of responses (resistant and susceptible) to bakanae disease. The results showed that general expression of glucanases, peroxidases and PR genes in both, resistant and susceptible, genotypes could represent the basic defence mechanism of rice against *F. fujikuroi*. What was found strikingly different between resistant and susceptible response is the modulation of WRKY transcriptional factors and MAPK cascades, which are involved in induction of different plant signalling pathways [46, 49]. Thus, 22 WRKY genes and 7 MAP kinases were up-regulated in the resistant genotype. Moreover, we found some cytochrome P450 genes involved in production of defence-related metabolites highly expressed only in Selenio. The cytochrome P450 class of enzymes will be further studied to assess their role in the production of secondary metabolites, such as sakuranetin and momilactones [14, 80], involved in resistance mechanisms. Transcriptional and enzyme-related genes associated with induction of jasmonic acid pathway, hypersensitive response and response to chitin, as revealed by GO enrichment and KEGG analyses, create a scaffold of resistance in rice against *F. fujikuroi.* A recently published study about the chemical responses of rice to *F. fujikuroi* infection [80] confirms that the increase of gibberellic acid produced by the fungus in the susceptible cultivar restricts jasmonic acid signaling. On the contrary, the level of jasmonic acid is stable and not impaired by pathogen inoculation in the resistant cultivar. Moreover, the susceptible genotype specifically activated chitinase genes and gibberellin-metabolic genes which, together with salicylic acid signalling and gibberellin metabolic pathway found in Dorella, represent the key factors in rice susceptibility towards bakanae disease.

The DEGs and GOs found in this study suggest that *F. fujikuroi* may behave differently with respect to genotype; e.g. in Dorella it acts systemically as a necrotroph destroying the plant cells, but in Selenio it is locally present, though at lower cell concentration, without damaging the plants and triggering hypersensitive response. This provides important information for a better understanding of molecular and cellular processes during *F. fujikuroi* infection and bakanae resistant pathways and offers a framework for development of bakanae resistant rice germplasm.

## Methods

### Screening of rice cultivars against bakanae disease in greenhouse

*F. fujikuroi* strain I1.3 isolated and characterized from Italian bakanae-infected rice [21, 81] was cultured together in potato dextrose broth (PDB, Merck KGaA) for 10 days in darkness at 20 °C. The strain was filtered through sterile cheese cloth to obtain a final spore concentration of 10^6^ mL^−1^. Twelve rice commercial genotypes were screened against bakanae disease: Leto, Dorella, Volano, Thaibonnet, Vialone Nano, Augusto, Eurosis, Carnaroli, Centauro, Gange, Selenio, and Baldo. The susceptible rice genotype Galileo was also included as a control. Seed lots of all rice cultivars were provided by the Rice Research Unit of Consiglio per la Ricerca e la Sperimentazione in Agricoltura. Rice seeds were surface-disinfected in 1 % sodium hypochlorite for 2 min and rinsed in sterile distilled water. A total of 120 seeds were soaked in 100 mL spore suspension and shaken for 30 min at room temperature, while control seeds for each genotype were soaked in sterile distilled water.

Seeds were sown in plastic pots with three biological replicates (40 seeds per pot) per each control (mock) and bakanae inoculated treatment. The plants were maintained in greenhouse conditions (25 °C day:17 °C night; watering 3 times per day). Disease symptoms were evaluated at 3 wpg using a bakanae disease symptomatic scale [22]: symptomless plants (0), plants with chlorotic, narrow leaves and delayed growth (25), plants with thin and elongated internodes (50), plants with crown necrosis (75), and dead plants (100). Germination rate was also evaluated. The screening of the rice genotypes was performed twice.

### Quantification of *F. fujikuroi* in selected rice cultivars

Deletion of six nucleotide onto Elongation factor 1α sequence, observed by Amatulli et al. [81] on *F. fujikuroi* was used for a TaqMan specific probe design (5′-[FAM]-TTGTCACGTGTCAAACTAAACATTCGAC-[TAMRA]-3′). Specific primers FjnsF (5′-ATGGGCGCGTTTTGCCCTTT-3′) and FjnsR (5′-GGCGTACTTGAAGGAACCCT-3′) were constructed using PerlPrimer software in order to obtain a 117 bp-amplicon. Real-time PCR was performed in a total volume of 25 μL by mixing 12.5 μL TaqMan Universal PCR Master Mix (Applied Biosystems), 0.56 μL each primer (10 μM), 0.5 μL TaqMan probe (5 μM), and 0.5 μL fungal DNA template. The reaction was performed on Applied Biosystems Stepone Plus RealTime PCR with the following conditions: initial denaturation at 95 °C for 15 min followed by 40 cycles of denaturation (30 s at 95 °C), annealing and extension (60 s at 64 °C).

### RNA extraction and Illumina GAIIx Sequencing

Total RNA was extracted from Selenio and Dorella at two infection time points (1 and 3 wpg) with RNeasy plant mini kit (Qiagen) following the manufacturer’s instructions. Three micrograms total RNA were used to construct libraries by TruSeq RNA sample preparation kit (FC-122-1001) according to the manufacturer’s instructions. Libraries were PCR-amplified in 15 cycles, purified, and size-selected for ~300 bp on a 2 % low range ultra-agarose gel (BIO-RAD). The Agilent 2100 bioanalyzer was used to check the quality of RNA and libraries. Single-end sequencing (51 bp) was conducted on an Illumina Genome Analyser (GAIIx) running two samples per lane.

### Bioinformatics

#### Differentially expressed genes

For DEG identification, DESEq2 1.2.5 R package was run with parametric fit and betaPrior parameter set to TRUE. Independent filtering was enabled [19, 20]. A False Discovery Rate (FDR) of 0.05 and a fold change of 2 were set as thresholds for DEG calling, as previously described [14, 82]. The list of all DEGs is provided (Additional file 3: Table S3) to allow any further DEG subsetting based on different FDR or fold change.

#### GO enrichment analyses

GO enrichment analyses were conducted with the goseq bioconductor package version 1.14.0. Goseq was specifically designed to contain length-derived bias which may affect RNA-seq data [51]. As rice databases are not yet covered by goseq built-in databases, transcript lengths were retrieved with BiomaRt queries (Ensembl plants; Nipponbare; Oryza_sativa.IRGSP-1.0.20) and median length of transcripts for each rice locus were obtained by parsing cDNA lengths with R custom scripts. A FDR cutoff of 0.1 was used for GO enrichments. As only GOSLIM terms are available in current ensemble plants Oryza_sativa IRGSP releases, full GO terms for each gene were retrieved by fastannotator queries using default parameters [83].

#### Other bioinformatic analyses

Unless otherwise stated, further graphical outputs were generated with custom R and Python scripts. The Bioconductor package pathview version 1.2.3 [75] was used to generate relevant KEGG pathway pictures incorporating color-coded expression values. Pathview parameters were set as default and the limit parameter was set as: limit = list (gene = 5, cpd = 1).

#### Production and analysis of gibberellin A_3_

Two hundred mg fresh rice material collected within three biological replicates were transferred in centrifuge tubes and 1 mL of extraction solution (CH_3_OH 80 % acidified with CH_3_CHOOH 0.1 %) was added. Samples were frozen with liquid nitrogen and homogenized with TissueLyser (Qiagen), then they were shaken at 4 °C in the dark overnight. At last, samples were centrifuged at 15,000 rpm and 4 °C for 2 min, and the supernatant was analysed by HPLC-MS/MS using a 1260 Agilent Technologies system consisting of a binary pump and a vacuum degasser. Aliquots (10 μL) were injected on a Luna Phenyl-Hexyl column (150 × 2 mm 3 μ, Phenomenex) under a flow of 200 μL/min. Solvent A was H_2_O with 0.1 % HCOOH, solvent B was CH_3_CN. GA_3_ was eluted in isocratic conditions 60:40 v/v for 10 min.

Using an electrospray (ESI) ion source operating in negative ion mode, samples were introduced into a triple-quadrupole mass spectrometer (Varian 310-MS TQ Mass Spectrometer), according to Sicilano et al. [80]. Two transitions were selected [345 > 239 (CE 14) and 345 > 143 (CE 30)]; the first transition was used for quantification and the second was the monitoring transition. The collision gas (Ar) pressure was set at 2 mbar for all experiments.

### qRT-PCR for expression of selected genes

The validation of the RNA-seq technique was performed by quantitative RT-PCR through monitoring the expression levels of seven selected transcripts (Additional file 17: Table S17). Total RNA was DNase treated using TURBO DNase (Ambion) according to the manufacturer’s protocol, and then 500 ng total RNA was reverse-transcribed using the High capacity cDNA reverse transcription kit (Applied Biosystems). PCR amplifications were carried out in an iCycler (BIO-RAD) containing 1 μL cDNA, 10 μL SsoFast™ EvaGreen® Supermix 2× (BIO-RAD), 0.25 μM each primer in a total volume of 20 μL. The rice EF1α gene [24] was used as a control for the constitutive expression. Primers for selected transcripts (Additional file 17: Table S17) were designed by Primer3 software (http://frodo.wi.mit.edu/primer3/). The expression ratio was calculated using the 2^−ΔΔCT^ method [84] showing the average of three technical replicates and three biological replicates.

### Statistical analysis

Data from the experiments of quantification of *F. fujikuroi*, production of gibberellin A_3_ and qRT-PCR were submitted to analysis of variance (ANOVA) by using the Statistical Package for Social Science (SPSS, IBM, Chicago, IL, USA) version 17.0. The statistical significance was judged at *P* < 0.05.

## Additional files

Additional file 1: Table S1. Raw and mapped reads details. Samples are referred to different Selenio (S) and Dorella (D) conditions: disease status (bakanae or mock inoculated), biological replicates (R1, R2, and R3), and growth stages (one week-W1 and three weeks-W3 post germination). (DOCX 15 kb) Additional file 2: Table S2. Pearson correlation coefficients of expression levels in different Dorella (D) and Selenio (S) conditions (bakanae or mock inoculated), biological replicates (R1, R2, and R3), and growth stages; one week (W1) and three weeks (W3) post germination. (DOCX 17 kb) Additional file 3: Table S3. Expression values of all detected transcribed genes and non-coding RNAs reported as DESeq-normalized read counts and log2 fold changes. (XLSX 41091 kb) Additional file 4: Table S4. Up-regulated and down-regulated cytochrome P450 genes. (XLSX 22 kb) Additional file 5: Table S5. Up-regulated and down-regulated PR genes. (XLSX 20 kb) Additional file 6: Table S6. Up-regulated and down-regulated glycoside hydrolase genes. (XLSX 20 kb) Additional file 7: Table S7. Up-regulated and down-regulated chitinase genes. (XLSX 17 kb) Additional file 8: Table S8. Up-regulated and down-regulated peroxidase genes. (XLSX 21 kb) Additional file 9: Table S9. Up-regulated and down-regulated germin-like protein genes. (XLSX 17 kb) Additional file 10: Table S10. Up-regulated and down-regulated MAP kinase genes. (XLSX 15 kb) Additional file 11: Table S11. Up-regulated and down-regulated WRKY genes. (XLSX 22 kb) Additional file 12: Table S12. List of the DEGs in the enriched GO term ‘plant-type hypersensitive response’ (GO:0009626) in Selenio and Dorella in 3 weeks post germination. (DOCX 15 kb) Additional file 13: Table S13. List of the DEGs in the enriched GO term ‘jasmonic acid biosynthetic process (GO:0009695) in Selenio and Dorella in 3 weeks post germination. (DOCX 14 kb) Additional file 14: Table S14. List of the DEGs in the enriched GO term ‘response to chitin’ (GO:0010200) in Selenio and Dorella in 3 weeks post germination. (DOCX 16 kb) Additional file 15: Table S15. List of the DEGs in the enriched GO term ‘response to salicylic acid stimulus’ (GO:0009751) in Selenio and Dorella in 3 weeks post germination. (DOCX 16 kb) Additional file 16: Table S16. List of the DEGs in the enriched GO term ‘gibberellin metabolic process’ (GO:0009685) in Selenio and Dorella in 3 weeks post germination. (DOCX 15 kb) Additional file 17: Table S17. List of primers used in quantitative RT-PCR for validation of RNA-Seq analysis. (DOCX 15 kb)
