# Supplementary material for: Comparative transcriptome profiling of resistant and susceptible rice genotypes in response to the seedborne pathogen Fusarium fujikuroi
Source: BMC Genomics. 2016 Aug 11;17:608. doi: 10.1186/s12864-016-2925-6 (PMC4981969; doi:10.1186/s12864-016-2925-6)
Supplement: Additional file 1: Table S1. — Raw and mapped reads details. Samples are referred to different Selenio (S) and Dorella (D) conditions: disease status (bakanae or mock inoculated), biological replicates (R1, R2, and R3), and growth stages (one week-W1 and three weeks-W3 post germination). (DOCX 15 kb) [file 12864_2016_2925_MOESM1_ESM.docx]

**Table S1**. Raw and mapped reads details. Samples are referred to different Selenio (S) and Dorella (D) conditions: disease status (bakanae or mock inoculated), biological replicates (R1, R2, and R3), and growth stages (one week-W1 and three weeks-W3 post germination)

| Sample | Illumina passed-filter reads | Contaminant-free  reads | Mapped reads | Reads mapped in unique regions |
| --- | --- | --- | --- | --- |
| S-R1-mock-W1 | 16462873 | 16216970 | 15836825 | 12964732 |
| S-R2-mock-W1 | 12550004 | 11903879 | 11295930 | 10742291 |
| S-R3-mock-W1 | 13054214 | 12791524 | 12459105 | 11876523 |
| S-R1-bakanae-W1 | 16115175 | 15879860 | 15513917 | 14759736 |
| S-R2-bakanae-W1 | 21619562 | 21327153 | 20871898 | 19890734 |
| S-R3-bakanae-W1 | 31631602 | 31159636 | 30575591 | 28997168 |
| D-R1-mock-W1 | 17103046 | 16862525 | 16415844 | 15670647 |
| D-R2-mock-W1 | 23841605 | 23528861 | 22965302 | 21895540 |
| D-R3-mock-W1 | 19780133 | 19363562 | 18533334 | 17684819 |
| D-R1-bakanae-W1 | 23011223 | 22497335 | 21944502 | 20966976 |
| D-R2-bakanae-W1 | 13697534 | 13490885 | 13102207 | 12525230 |
| D-R3-bakanae-W1 | 22627900 | 22193069 | 21659819 | 20721124 |
| S-R1-mock-W3 | 18893205 | 18651570 | 18281877 | 17325574 |
| S-R2-mock-W3 | 14600431 | 13887976 | 13251570 | 12415301 |
| S-R3-mock-W3 | 20364976 | 20013160 | 19607673 | 18590737 |
| S-R1-bakanae-W3 | 23336998 | 23052717 | 22638913 | 21549469 |
| S-R2-bakanae-W3 | 13586658 | 13463695 | 13186526 | 12552507 |
| S-R3-bakanae-W3 | 8335049 | 8249716 | 8045749 | 7668106 |
| D-R1-mock-W3 | 19925979 | 19723770 | 19252197 | 18351920 |
| D-R2-mock-W3 | 11931585 | 11804936 | 11498991 | 10975329 |
| D-R3-mock-W3 | 14490303 | 14242789 | 13650467 | 12938066 |
| D-R1-bakanae-W3 | 15442307 | 15132416 | 14711989 | 13978963 |
| D-R2-bakanae-W3 | 17532256 | 17284836 | 16797952 | 15897113 |
| D-R3-bakanae-W3 | 16797308 | 16538073 | 16087017 | 15268306 |
