# Supplementary material for: Comparative transcriptome profiling of resistant and susceptible rice genotypes in response to the seedborne pathogen Fusarium fujikuroi
Source: BMC Genomics. 2016 Aug 11;17:608. doi: 10.1186/s12864-016-2925-6 (PMC4981969; doi:10.1186/s12864-016-2925-6)
Supplement: Additional file 2: Table S2. — Pearson correlation coefficients of expression levels in different Dorella (D) and Selenio (S) conditions (bakanae or mock inoculated), biological replicates (R1, R2, and R3), and growth stages; one week (W1) and three weeks (W3) post germination. (DOCX 17 kb) [file 12864_2016_2925_MOESM2_ESM.docx]

**Table S2.** Pearson correlation coefficients of expression levels in different Dorella (D) and Selenio (S) conditions (bakanae or mock inoculated), biological replicates (R1, R2, and R3), and growth stages; one week (W1) and three weeks (W3) post germination

|  | S_R1_  bakanae_W1 | S_R2_  bakanae_W1 | S_R3_  bakanae_W1 | S_R1_  bakanae_W3 | S_R2_  bakanae_W3 | S_R3_  bakanae_W3 | S_R1_mock_W1 | S_R2_mock_W1 | S_R3_mock_W1 | S_R1_mock_W3 | S_R2_mock_W3 | S_R3_mock_W3 |
| --- | --- | --- | --- | --- | --- | --- | --- | --- | --- | --- | --- | --- |
| D_R1_bakanae_W1 | 0.9695 | 0.9673 | 0.9684 | 0.8995 | 0.8999 | 0.8953 | 0.9573 | 0.9314 | 0.9547 | 0.9062 | 0.9089 | 0.9107 |
| D_R2_bakanae_W1 | 0.9751 | 0.9759 | 0.9750 | 0.9000 | 0.9000 | 0.8935 | 0.9666 | 0.9419 | 0.9648 | 0.9102 | 0.9139 | 0.9151 |
| D_R3_bakanae_W1 | 0.9679 | 0.9665 | 0.9668 | 0.9022 | 0.9035 | 0.8990 | 0.9575 | 0.9328 | 0.9550 | 0.9036 | 0.9061 | 0.9081 |
| D_R1_bakanae_W3 | 0.7266 | 0.7531 | 0.7329 | 0.6675 | 0.6850 | 0.6718 | 0.7414 | 0.7484 | 0.7524 | 0.6744 | 0.6853 | 0.6822 |
| D_R2_bakanae_W3 | 0.6591 | 0.6786 | 0.6656 | 0.6562 | 0.6718 | 0.6600 | 0.6620 | 0.6611 | 0.6714 | 0.6764 | 0.6851 | 0.6818 |
| D_R3_bakanae_W3 | 0.7099 | 0.7359 | 0.7161 | 0.6528 | 0.6707 | 0.6581 | 0.7242 | 0.7337 | 0.7356 | 0.6614 | 0.6721 | 0.6682 |
| D_R1_mock_W1 | 0.9861 | 0.9901 | 0.9866 | 0.8736 | 0.8745 | 0.8670 | 0.9878 | 0.9757 | 0.9881 | 0.8907 | 0.8965 | 0.8963 |
| D_R2_mock_W1 | 0.9907 | 0.9960 | 0.9924 | 0.8745 | 0.8727 | 0.8637 | 0.9948 | 0.9838 | 0.9956 | 0.9012 | 0.9080 | 0.9066 |
| D_R3_mock_W1 | 0.9899 | 0.9959 | 0.9919 | 0.8709 | 0.8694 | 0.8599 | 0.9947 | 0.9858 | 0.9962 | 0.8991 | 0.9061 | 0.9044 |
| D_R1_mock_W3 | 0.6226 | 0.6414 | 0.6270 | 0.7011 | 0.7151 | 0.7067 | 0.6304 | 0.6076 | 0.6337 | 0.6402 | 0.6420 | 0.6471 |
| D_R2_mock_W3 | 0.7386 | 0.7346 | 0.7383 | 0.8668 | 0.8785 | 0.8769 | 0.7300 | 0.6870 | 0.7228 | 0.7831 | 0.7828 | 0.7886 |
| D_R3_mock_W3 | 0.7577 | 0.7542 | 0.7577 | 0.8814 | 0.8917 | 0.8889 | 0.7496 | 0.7069 | 0.7429 | 0.8022 | 0.8022 | 0.8077 |
| S_R1_bakanae_W1 | 1.0000 | 0.9953 | 0.9996 | 0.8985 | 0.8940 | 0.8879 | 0.9939 | 0.9787 | 0.9911 | 0.9302 | 0.9341 | 0.9340 |
| S_R2_bakanae_W1 | 0.9953 | 1.0000 | 0.9972 | 0.8796 | 0.8774 | 0.8686 | 0.9965 | 0.9837 | 0.9971 | 0.9092 | 0.9149 | 0.9148 |
| S_R3_bakanae_W1 | 0.9996 | 0.9972 | 1.0000 | 0.8958 | 0.8917 | 0.8848 | 0.9951 | 0.9804 | 0.9932 | 0.9279 | 0.9322 | 0.9322 |
| S_R1_bakanae_W3 | 0.8985 | 0.8796 | 0.8958 | 1.0000 | 0.9984 | 0.9980 | 0.8764 | 0.8313 | 0.8657 | 0.9723 | 0.9701 | 0.9736 |
| S_R2_bakanae_W3 | 0.8940 | 0.8774 | 0.8917 | 0.9984 | 1.0000 | 0.9987 | 0.8732 | 0.8281 | 0.8633 | 0.9654 | 0.9637 | 0.9675 |
| S_R3_bakanae_W3 | 0.8879 | 0.8686 | 0.8848 | 0.9980 | 0.9987 | 1.0000 | 0.8651 | 0.8185 | 0.8540 | 0.9633 | 0.9605 | 0.9645 |
| S_R1_mock_W1 | 0.9939 | 0.9965 | 0.9951 | 0.8764 | 0.8732 | 0.8651 | 1.0000 | 0.9910 | 0.9987 | 0.9053 | 0.9115 | 0.9100 |
| S_R2_mock_W1 | 0.9787 | 0.9837 | 0.9804 | 0.8313 | 0.8281 | 0.8185 | 0.9910 | 1.0000 | 0.9927 | 0.8720 | 0.8805 | 0.8755 |
| S_R3_mock_W1 | 0.9911 | 0.9971 | 0.9932 | 0.8657 | 0.8633 | 0.8540 | 0.9987 | 0.9927 | 1.0000 | 0.8963 | 0.9033 | 0.9015 |
| S_R1_mock_W3 | 0.9302 | 0.9092 | 0.9279 | 0.9723 | 0.9654 | 0.9633 | 0.9053 | 0.8720 | 0.8963 | 1.0000 | 0.9993 | 0.9992 |
| S_R2_mock_W3 | 0.9341 | 0.9149 | 0.9322 | 0.9701 | 0.9637 | 0.9605 | 0.9115 | 0.8805 | 0.9033 | 0.9993 | 1.0000 | 0.9990 |
| S_R3_mock_W3 | 0.9340 | 0.9148 | 0.9322 | 0.9736 | 0.9675 | 0.9645 | 0.9100 | 0.8755 | 0.9015 | 0.9992 | 0.9990 | 1.0000 |

|  | D_R1_  bakanae_W1 | D_R2_  bakanae_W1 | D_R3_  bakanae_W1 | D_R1_  bakanae_W3 | D_R2_  bakanae_W3 | D_R3_  bakanae_W3 | D_R1_mock_W1 | D_R2_mock_W1 | D_R3_mock_W1 | D_R1_mock_W3 | D_R2_mock_W3 | D_R3_mock_W3 |
| --- | --- | --- | --- | --- | --- | --- | --- | --- | --- | --- | --- | --- |
| D_R1_bakanae_W1 | 1.0000 | 0.9979 | 0.9969 | 0.7545 | 0.6849 | 0.7377 | 0.9776 | 0.9694 | 0.9665 | 0.6624 | 0.7826 | 0.7965 |
| D_R2_bakanae_W1 | 0.9979 | 1.0000 | 0.9948 | 0.7601 | 0.6901 | 0.7424 | 0.9818 | 0.9781 | 0.9756 | 0.6665 | 0.7802 | 0.7961 |
| D_R3_bakanae_W1 | 0.9969 | 0.9948 | 1.0000 | 0.7646 | 0.6887 | 0.7484 | 0.9810 | 0.9685 | 0.9661 | 0.6834 | 0.7946 | 0.8075 |
| D_R1_bakanae_W3 | 0.7545 | 0.7601 | 0.7646 | 1.0000 | 0.9581 | 0.9983 | 0.7729 | 0.7583 | 0.7624 | 0.8513 | 0.6707 | 0.6750 |
| D_R2_bakanae_W3 | 0.6849 | 0.6901 | 0.6887 | 0.9581 | 1.0000 | 0.9617 | 0.6872 | 0.6794 | 0.6832 | 0.8344 | 0.6455 | 0.6501 |
| D_R3_bakanae_W3 | 0.7377 | 0.7424 | 0.7484 | 0.9983 | 0.9617 | 1.0000 | 0.7569 | 0.7408 | 0.7456 | 0.8495 | 0.6585 | 0.6618 |
| D_R1_mock_W1 | 0.9776 | 0.9818 | 0.9810 | 0.7729 | 0.6872 | 0.7569 | 1.0000 | 0.9941 | 0.9937 | 0.6601 | 0.7604 | 0.7759 |
| D_R2_mock_W1 | 0.9694 | 0.9781 | 0.9685 | 0.7583 | 0.6794 | 0.7408 | 0.9941 | 1.0000 | 0.9997 | 0.6400 | 0.7424 | 0.7614 |
| D_R3_mock_W1 | 0.9665 | 0.9756 | 0.9661 | 0.7624 | 0.6832 | 0.7456 | 0.9937 | 0.9997 | 1.0000 | 0.6410 | 0.7387 | 0.7579 |
| D_R1_mock_W3 | 0.6624 | 0.6665 | 0.6834 | 0.8513 | 0.8344 | 0.8495 | 0.6601 | 0.6400 | 0.6410 | 1.0000 | 0.7510 | 0.7535 |
| D_R2_mock_W3 | 0.7826 | 0.7802 | 0.7946 | 0.6707 | 0.6455 | 0.6585 | 0.7604 | 0.7424 | 0.7387 | 0.7510 | 1.0000 | 0.9979 |
| D_R3_mock_W3 | 0.7965 | 0.7961 | 0.8075 | 0.6750 | 0.6501 | 0.6618 | 0.7759 | 0.7614 | 0.7579 | 0.7535 | 0.9979 | 1.0000 |
| S_R1_bakanae_W1 | 0.9695 | 0.9751 | 0.9679 | 0.7266 | 0.6591 | 0.7099 | 0.9861 | 0.9907 | 0.9899 | 0.6226 | 0.7386 | 0.7577 |
| S_R2_bakanae_W1 | 0.9673 | 0.9759 | 0.9665 | 0.7531 | 0.6786 | 0.7359 | 0.9901 | 0.9960 | 0.9959 | 0.6414 | 0.7346 | 0.7542 |
| S_R3_bakanae_W1 | 0.9684 | 0.9750 | 0.9668 | 0.7329 | 0.6656 | 0.7161 | 0.9866 | 0.9924 | 0.9919 | 0.6270 | 0.7383 | 0.7577 |
| S_R1_bakanae_W3 | 0.8995 | 0.9000 | 0.9022 | 0.6675 | 0.6562 | 0.6528 | 0.8736 | 0.8745 | 0.8709 | 0.7011 | 0.8668 | 0.8814 |
| S_R2_bakanae_W3 | 0.8999 | 0.9000 | 0.9035 | 0.6850 | 0.6718 | 0.6707 | 0.8745 | 0.8727 | 0.8694 | 0.7151 | 0.8785 | 0.8917 |
| S_R3_bakanae_W3 | 0.8953 | 0.8935 | 0.8990 | 0.6718 | 0.6600 | 0.6581 | 0.8670 | 0.8637 | 0.8599 | 0.7067 | 0.8769 | 0.8889 |
| S_R1_mock_W1 | 0.9573 | 0.9666 | 0.9575 | 0.7414 | 0.6620 | 0.7242 | 0.9878 | 0.9948 | 0.9947 | 0.6304 | 0.7300 | 0.7496 |
| S_R2_mock_W1 | 0.9314 | 0.9419 | 0.9328 | 0.7484 | 0.6611 | 0.7337 | 0.9757 | 0.9838 | 0.9858 | 0.6076 | 0.6870 | 0.7069 |
| S_R3_mock_W1 | 0.9547 | 0.9648 | 0.9550 | 0.7524 | 0.6714 | 0.7356 | 0.9881 | 0.9956 | 0.9962 | 0.6337 | 0.7228 | 0.7429 |
| S_R1_mock_W3 | 0.9062 | 0.9102 | 0.9036 | 0.6744 | 0.6764 | 0.6614 | 0.8907 | 0.9012 | 0.8991 | 0.6402 | 0.7831 | 0.8022 |
| S_R2_mock_W3 | 0.9089 | 0.9139 | 0.9061 | 0.6853 | 0.6851 | 0.6721 | 0.8965 | 0.9080 | 0.9061 | 0.6420 | 0.7828 | 0.8022 |
| S_R3_mock_W3 | 0.9107 | 0.9151 | 0.9081 | 0.6822 | 0.6818 | 0.6682 | 0.8963 | 0.9066 | 0.9044 | 0.6471 | 0.7886 | 0.8077 |
