# Supplementary material for: Comparative transcriptome profiling of resistant and susceptible rice genotypes in response to the seedborne pathogen Fusarium fujikuroi
Source: BMC Genomics. 2016 Aug 11;17:608. doi: 10.1186/s12864-016-2925-6 (PMC4981969; doi:10.1186/s12864-016-2925-6)
Supplement: Additional file 12: Table S12. — List of the DEGs in the enriched GO term ‘plant-type hypersensitive response’ (GO:0009626) in Selenio and Dorella in 3 weeks post germination. (DOCX 15 kb) [file 12864_2016_2925_MOESM12_ESM.docx]

|  |  |  | **Selenio** | | | | **Dorella** | | | |
| --- | --- | --- | --- | --- | --- | --- | --- | --- | --- | --- |
| **id** | **RAP-DP annotation** | **Other annotations** | **baseMean** | **log2FC** | **FDR** | **Included in DEGs** | **baseMean** | **log2FC** | **FDR** | **Included in DEGs** |
| Os09g0378700 | Similar to ubiquitin-protein ligase | Putative Avr9/Cf-9 rapidly elicited protein 276 | 55,36 | 3,70 | 2,90E-39 | YES | 266,66 | -3,44 | NA | NO |
| Os03g0140900 | Conserved hypothetical protein | Os03g0140900 protein | 5,33 | 2,31 | 7,23E-06 | YES | 8,41 | -0,10 | 0,91 | NO |
| Os01g0748900 | Membrane attack complex component/perforin/complement C9 family protein | Putative uncharacterized protein P0481E12.33 | 2391,59 | 2,27 | 0 | YES | 2218,36 | -2,23 | 1,35E-18 | YES |
| Os02g0605900 | Similar to Chitinase (EC 3.2.1.14) A | Chitinase 6 | 4739,63 | 2,26 | 2,81E-305 | YES | 6745,48 | -3,67 | 5,55E-11 | YES |
| Os05g0135800 | Similar to Pto kinase interactor 1 | Putative Pto kinase interactor 1 | 4099,32 | 1,39 | 0 | YES | 3124,56 | -0,44 | 0,24 | NO |
| Os02g0521300 | C2 domain containing protein | Putative copine III | 1292,26 | 1,06 | 4,51E-69 | YES | 2022,98 | -1,04 | 1,83E-05 | YES |
| Os07g0673200 | Similar to F22D16.14 protein (RING finger family protein) | Probable E3 ubiquitin-protein ligase BAH1-like 2 | 270,14 | 1,03 | 3,02E-19 | YES | 359,82 | -1,52 | 0,00 | YES |
| Os05g0373400 | Ribonucleoprotein, BRUNO-like domain containing protein | Os05g0373400 protein | 143,49 | 1,01 | 2,09E-11 | YES | 160,21 | -0,02 | 0,96 | NO |
| Os11g0592200 | Similar to Chitin-binding allergen Bra r 2 | Pathogenesis-related protein; Win1 | 17,72 | -0,40 | 0,39 | NO | 178,26 | 2,18 | 0,00 | YES |
| Os03g0758250 | Hypothetical gene |  | 18,40 | -0,06 | 0,90 | NO | 18,86 | -1,13 | 0,03 | YES |
| Os03g0650900 | Zinc finger, RING/FYVE/PHD-type domain containing protein | Probable E3 ubiquitin-protein ligase BAH1-like 1 | 32,56 | 0,28 | 0,44 | NO | 174,90 | -1,23 | 0,01 | YES |
| Os02g0234300 | Armadillo-like helical domain containing protein | U-box domain-containing protein 4 | 1001,19 | 0,46 | 1,32E-13 | NO | 804,99 | -1,32 | 0 | YES |
| Os03g0223200 | Similar to Cinnamyl alcohol dehydrogenase | Probable cinnamyl alcohol dehydrogenase 9 | 4,86 | -0,26 | 0,73 | NO | 9,62 | -1,61 | 0,02 | YES |

**Table S12.** List of the DEGs in the enriched GO term ‘plant-type hypersensitive response’ (GO:0009626) in Selenio and Dorella in 3 weeks post germination
