# Supplementary material for: Comparative transcriptome profiling of resistant and susceptible rice genotypes in response to the seedborne pathogen Fusarium fujikuroi
Source: BMC Genomics. 2016 Aug 11;17:608. doi: 10.1186/s12864-016-2925-6 (PMC4981969; doi:10.1186/s12864-016-2925-6)
Supplement: Additional file 13: Table S13. — List of the DEGs in the enriched GO term ‘jasmonic acid biosynthetic process (GO:0009695) in Selenio and Dorella in 3 weeks post germination. (DOCX 14 kb) [file 12864_2016_2925_MOESM13_ESM.docx]

**Table S13.** List of the DEGs in the enriched GO term ‘jasmonic acid biosynthetic process (GO:0009695) in Selenio and Dorella in 3 weeks post germination

|  |  |  | **Selenio** | | | | **Dorella** | | | |
| --- | --- | --- | --- | --- | --- | --- | --- | --- | --- | --- |
| **id** | **RAP-DP annotation** | **Other annotations** | **baseMean** | **log2FC** | **FDR** | **Included in DEGS** | **baseMean** | **log2FC** | **FDR** | **Included in DEGS** |
| Os11g0299300 | Lipase, class 3 family protein | Lipase class 3 family protein | 46,97 | 2,54 | 1,06E-24 | YES | 59,77 | -0,67 | 0,41 | NO |
| Os06g0216300 | Similar to 12-oxophytodienoic acid  reductase | | 2,40 | 0,19 | 4,30E-36 | YES | 507,20 | -4,36 | 1,65E-13 | YES |
| Os10g0457600 | Similar to Acetyl-CoA C-acyltransferase | 3-ketoacyl-CoA thiolase 2 | 2186,92 | 2,20 | 6,64E-221 | YES | 5000,96 | -2,36 | 0,00 | YES |
| Os04g0447100 | Similar to Lipoxygenase | Putative lipoxygenase 5 | 1448,25 | 2,01 | 2,33E-112 | YES | 962,54 | -1,15 | NA | NO |
| Os03g0438100 | Similar to Allene oxide cyclase precursor | AOC; Allene oxide cyclase | 1257,13 | 1,50 | 7,18E-169 | YES | 1923,49 | -1,26 | 0,00 | YES |
| Os03g0132000 | Similar to 4-coumarate-CoA ligase-like protein | 4-coumarate--CoA ligase-like 4 | 504,13 | 1,40 | 1,86E-79 | YES | 567,30 | -1,03 | 0,00 | YES |
| Os01g0901500 | Similar to 4-coumarate--CoA ligase-like 5 | 4-coumarate--CoA ligase-like 5 | 160,45 | 0,02 | 0,94 | NO | 189,74 | -1,34 | 0,05 | YES |
| Os03g0767000 | Similar to Allene oxide synthase | Allene oxide synthase 1, chloroplastic | 58,39 | 2,19 | 1,24E-23 | YES | 31,75 | -1,14 | 0,14 | NO |
