# Supplementary material for: Comparative transcriptome profiling of resistant and susceptible rice genotypes in response to the seedborne pathogen Fusarium fujikuroi
Source: BMC Genomics. 2016 Aug 11;17:608. doi: 10.1186/s12864-016-2925-6 (PMC4981969; doi:10.1186/s12864-016-2925-6)
Supplement: Additional file 14: Table S14. — List of the DEGs in the enriched GO term ‘response to chitin’ (GO:0010200) in Selenio and Dorella in 3 weeks post germination. (DOCX 16 kb) [file 12864_2016_2925_MOESM14_ESM.docx]

|  |  |  | **Selenio** | | | | **Dorella** | | | |
| --- | --- | --- | --- | --- | --- | --- | --- | --- | --- | --- |
| **id** | **RAP-DP annotation** | **Other annotations** | **baseMean** | **log2FC** | **FDR** | **Included in DEGS** | **baseMean** | **log2FC** | **FDR** | **Included in DEGS** |
| Os09g0526600 | Similar to Isoform 2 of Heat stress transcription factor B-2c | Heat stress transcription factor B-2c | 636,90 | 1,74 | 2,10E-173 | YES | 1181,90 | -3,19 | NA | NO |
| Os08g0437300 | Similar to Typical P-type R2R3 Myb protein | Putative typical P-type R2R3 Myb protein | 6,45 | -1,79 | 0,00 | YES | 12,03 | 2,52 | 0,00 | YES |
| Os05g0572000 | Pathogenesis-related transcriptional factor and ERF domain containing protein | Putative uncharacterized protein OJ1735_C10.9 | 94,79 | 1,00 | 7,92E-10 | YES | 205,56 | -3,20 | NA | NO |
| Os08g0546800 | Similar to Heat stress transcription factor B-2b | Heat stress transcription factor B-2b | 134,09 | 2,42 | 5,29E-31 | YES | 224,29 | -2,27 | NA | NO |
| Os11g0700500 | Similar to Snapdragon myb protein 305 homolog | Myb-related protein MYBAS1 | 72,48 | -2,48 | 4,83E-27 | YES | 59,03 | 0,44 | 0,60 | NO |
| Os08g0415600 | Similar to ubiquitin-protein ligase | Putative arm repeat protein | 63,90 | 1,51 | 1,20E-16 | YES | 139,54 | -2,34 | NA | NO |
| Os05g0420300 | Similar to transcriptional factor TINY | Putative uncharacterized protein P0426G01.15 | 8,35 | 1,92 | 0,00 | YES | 1,42 | -0,52 | 0,61 | NO |
| Os02g0540700 | Similar to Arm repeat-containing protein | Putative photoperiod responsive protein | 2894,13 | 0,53 | NA | NO | 1512,07 | -1,59 | 0,00 | YES |
| Os03g0240600 | Zinc finger, RING/FYVE/PHD-type domain containing protein | U-box containing E3 ligase | 429,16 | 2,70 | 5,76E-105 | YES | 280,56 | -2,93 | NA | NO |
| Os03g0745000 | Similar to Heat stress transcription factor A-2a | -- | 212,30 | 3,00 | 6,98E-109 | YES | 302,93 | -2,97 | NA | NO |
| Os01g0823900 | Arm repeat protein | Arm repeat protein | 161,28 | 0,70 | 4,12E-09 | NO | 413,75 | -1,31 | 0,00 | YES |
| Os03g0860100 | Similar to Ethylene-responsive transcription factor 2 | AP2 domain containing protein | 3,82 | 2,16 | 6,90E-05 | YES | 7,56 | -0,77 | 0,39 | NO |
| Os02g0176000 | Zinc finger, B-box domain containing protein | Putative uncharacterized protein OJ1077_E05.17 | 399,74 | -1,01 | 4,00E-27 | YES | 141,76 | 0,36 | 0,48 | NO |
| Os03g0723000 | GRAS transcription factor domain containing protein | GRAS family transcription factor containing protein | 52,30 | 1,76 | 1,37E-13 | YES | 67,75 | -2,03 | 0,00 | YES |
| Os04g0437300 | Zinc finger, RING/FYVE/PHD-type domain containing protein | OSJNBa0006B20.11 protein | 5,86 | 1,71 | 0,00 | YES | 5,50 | 0,99 | 0,20 | NO |
| Os04g0418500 | Similar to photoperiod responsive protein | OSJNBb0108J11.6 protein | 763,62 | 1,65 | 6,21E-132 | YES | 480,06 | -1,34 | 3,16E-08 | YES |
| Os02g0743700 | Similar to RING-H2 finger protein ATL1Q | Putative NEP1-interacting protein | 29,60 | -0,37 | 0,24 | NO | 132,02 | 1,84 | 0,02 | YES |
| Os02g0561900 | Similar to E3 ubiquitin-protein ligase EL5 | -- | 12,25 | 0,84 | 0,05 | NO | 10,19 | 1,52 | 0,04 | YES |
| Os02g0548700 | Similar to ubiquitin-protein ligase | Os02g0548700 protein | 285,11 | 2,69 | 3,06E-45 | YES | 705,15 | -0,87 | 0,16 | NO |
| Os01g0885900 | Similar to transcriptional factor TINY | AP2 domain-containing transcription factor-like protein | 73,61 | 2,64 | 7,98E-25 | YES | 22,04 | -2,82 | 3,1E-07 | YES |
| Os01g0755700 | Zinc finger, RING/FYVE/PHD-type domain containing protein | RING zinc finger protein-like | 18,13 | 0,48 | 0,20 | NO | 127,61 | -1,84 | 0,00 | YES |

**Table S14.** List of the DEGs in the enriched GO term ‘response to chitin’ (GO:0010200) in Selenio and Dorella in 3 weeks post germination
