# Supplementary material for: Comparative transcriptome profiling of resistant and susceptible rice genotypes in response to the seedborne pathogen Fusarium fujikuroi
Source: BMC Genomics. 2016 Aug 11;17:608. doi: 10.1186/s12864-016-2925-6 (PMC4981969; doi:10.1186/s12864-016-2925-6)
Supplement: Additional file 15: Table S15. — List of the DEGs in the enriched GO term ‘response to salicylic acid stimulus’ (GO:0009751) in Selenio and Dorella in 3 weeks post germination. (DOCX 16 kb) [file 12864_2016_2925_MOESM15_ESM.docx]

|  |  |  | | **Selenio** | | | | | **Dorella** | | | |
| --- | --- | --- | --- | --- | --- | --- | --- | --- | --- | --- | --- | --- |
| **id** | **RAP-DP annotation** | **Other annotations** | | **baseMean** | **log2FC** | **FDR** | **Included in DEGS** | **baseMean** | | **log2FC** | **FDR** | **Included in DEGS** |
| Os06g0158100 | Similar to WRKY transcription factor 63 | Transcription factor WRKY35 | | 173,2045 | 3,9674 | 2,03E-104 | YES | 48,0404 | | -3,3356 | 2,73E-14 | YES |
| Os11g0116200 | Similar to nonspecific lipid-transfer protein 3 | Non-specific lipid-transfer protein | | 337,0781 | 0,0701 | 0,615213291 | NO | 710,7785 | | 1,8819 | 5,54E-08 | YES |
| Os12g0115500 | Similar to nonspecific lipid-transfer protein 3 | Non-specific lipid-transfer protein | | 1088,7964 | -0,0024 | 0,98254762 | NO | 1835,4529 | | 2,1886 | 1,26E-27 | YES |
| Os08g0437300 | Similar to Typical P-type R2R3 Myb protein | Putative typical P-type R2R3 Myb protein | | 6,4523 | -1,7919 | 0,000922653 | YES | 12,0336 | | 2,52 | 0,00011756 | YES |
| Os08g0157600 | MYB transcription factor, Circadian clock | Os08g0157600 protein | | 51319,1352 | 0,1605 | 4,30544E-06 | NO | 20943,731 | | -2,382 | 0,000862633 | YES |
| Os08g0157500 | Similar to Caffeic acid 3-O-methyltransferase | Flavone 3'-O-methyltransferase 1 | | 752,6129 | -0,6409 | 1,63E-14 | NO | 826,3964 | | 2,1222 | 0,000438639 | YES |
| Os10g0415900 | Similar to General control of amino-acid synthesis 5-like 2 | Histone acetyltransferase GCN5 | | 222,5081 | 0,8333 | 5,77E-13 | NO | 348,2596 | | -1,8222 | 0,002023579 | YES |
| Os07g0673200 | Similar to F22D16.14 protein (RING finger family protein) | Probable E3 ubiquitin-protein ligase BAH1-like 2 | | 270,144 | 1,0256 | 3,02E-19 | YES | 359,8154 | | -1,522 | 0,003247708 | YES |
| Os12g0123200 | Glutathione S-transferase GST 7 | Glutathione S-transferase | | 71,0146 | -0,2938 | 0,245020657 | NO | 177,7079 | | 1,3406 | 0,037731231 | YES |
| Os04g0446200 | Similar to OSIGBa0140O07.13 protein | - | | 92,647 | -1,0235 | 1,59E-06 | YES | 73,0639 | | 1,4657 | 3,24194E-06 | YES |
| Os01g0524500 | Conserved hypothetical protein | - | | 201,3876 | -2,5517 | 2,35E-59 | YES | 208,7901 | | 1,5157 | 1,46E-15 | YES |
| Os03g0650900 | Zinc finger, RING/FYVE/PHD-type domain containing protein | Probable E3 ubiquitin-protein ligase BAH1-like 1 | | 32,5607 | 0,2752 | 0,444884998 | NO | 174,9033 | | -1,2263 | 0,011419697 | YES |
| Os04g0594100 | Similar to P-type R2R3 Myb protein | OSJNBa0009P12.32 protein | | 0,4474 | -0,0938 | 0,887807534 | NO | 3,3123 | | 2,1536 | 0,009957928 | YES |
| Os03g0126000 | Similar to Phosphorybosyl anthranilate transferase 1 | Anthranilate phosphoribosyltransferase, chloroplast, putative, expressed | | 480,4924 | 0,3181 | 5,7193E-06 | NO | 1306,4601 | | -1,5075 | 0,001434824 | YES |
| Os01g0285300 | Myb transcription factor domain containing protein | Putative MYB2; Putative transcription factor (Myb) | | 1,5399 | 0,6846 | NA | NO | 24,8081 | | 3,7345 | 3,91E-08 | YES |
| Os11g0700500 | Similar to Snapdragon myb protein 305 homolog | | Myb-related protein MYBAS1 | 72,4849 | -2,4752 | 4,83E-27 | YES | 59,0321 | | 0,438 | 0,598627486 | NO |

**Table S15**. List of the DEGs in the enriched GO term ‘response to salicylic acid stimulus’ (GO:0009751) in Selenio and Dorella in 3 weeks post germination
