# Supplementary material for: Comparative transcriptome profiling of resistant and susceptible rice genotypes in response to the seedborne pathogen Fusarium fujikuroi
Source: BMC Genomics. 2016 Aug 11;17:608. doi: 10.1186/s12864-016-2925-6 (PMC4981969; doi:10.1186/s12864-016-2925-6)
Supplement: Additional file 16: Table S16. — List of the DEGs in the enriched GO term ‘gibberellin metabolic process’ (GO:0009685) in Selenio and Dorella in 3 weeks post germination. (DOCX 15 kb) [file 12864_2016_2925_MOESM16_ESM.docx]

|  |  |  | **Selenio** | | | | | **Dorella** | | | |
| --- | --- | --- | --- | --- | --- | --- | --- | --- | --- | --- | --- |
| **id** | **RAP-DP annotation** | **Other annotations** | **baseMean** | **log2FC** | **FDR** | **Included in DEGS** | **baseMean** | | **log2FC** | **FDR** | **Included in DEGS** |
| Os11g0474800 | Similar to Isoform 2 of Stemar-13-ene synthase | Stemar-13-ene synthase | 32,9689 | -0,4486 | 0,126174466 | NO | 38,7877 | | 1,9882 | 3,69013E-05 | YES |
| Os05g0560900 | Similar to gibberellin 2-beta-dioxygenase | Putative GA2-oxidase | 0,2518 | -0,3542 | 0,449761565 | NO | 4,4527 | | 2,2003 | 0,004970734 | YES |
| Os06g0110000 | Similar to DWARF3 (Fragment) | Putative cytochrome P450 DWARF3 | 19,4401 | -0,1123 | 0,844526472 | NO | 77,7393 | | 1,6489 | 0,033352302 | YES |
| Os06g0649000 | Similar to WRKY transcription factor 28 | Os06g0649000 protein | 1328,9589 | 3,3016 | 0 | YES | 514,504 | | -1,4001 | 0,001359723 | YES |
| Os02g0570900 | Similar to Ent-copalyl diphosphate synthase 2 | - | 3,6574 | 0,379 | NA | NO | 27,5575 | | -2,4258 | 0,000134298 | YES |
| Os02g0571000 | Hypothetical protein | - | 13,3123 | 0,6761 | 0,1337296 | NO | 46,0924 | | -1,8772 | 0,000771706 | YES |
| Os02g0181300 | Similar to WRKY transcription factor | - | 8762,5815 | 2,028 | 0 | YES | 11631,1512 | | -1,4659 | 0,000314446 | YES |
| Os02g0181200 | Hypothetical protein | - | 2580,889 | 2,4188 | 1,72E-203 | YES | 3205,7184 | | -2,155 | 1,0659E-07 | YES |
| Os03g0856700 | Gibberellin 20 oxidase 1 (EC 1.14.11.-) (Os20ox) | Gibberellin 20 oxidase 1 | 266,677 | -1,6533 | 6,82E-25 | YES | 46,0772 | | 1,7802 | 0,002315223 | YES |
| Os04g0178300 | Similar to Isoform 3 of Syn-copalyl diphosphate synthase | Syn-copalyl diphosphate synthase | 34,8037 | -0,4527 | 0,157632495 | NO | 50,6628 | | 2,2549 | 5,79051E-06 | YES |
| Os01g0209700 | Similar to GA 2-oxidase 5 | Putative GA 2-oxidase 5 | 20,3962 | 1,1954 | 0,001797327 | YES | 48,0996 | | -1,8638 | 0,001519946 | YES |
| Os02g0571100 | Terpenoid synthase domain containing protein | Ent-copalyl diphosphate synthase 2 | 119,7629 | 0,5651 | 4,07E-05 | NO | 305,3474 | | -1,3833 | 0,002861753 | YES |

**Table S16**. List of the DEGs in the enriched GO term ‘gibberellin metabolic process’ (GO:0009685) in Selenio and Dorella in 3 weeks post germination
