# Supplementary material for: Comparative transcriptome profiling of resistant and susceptible rice genotypes in response to the seedborne pathogen Fusarium fujikuroi
Source: BMC Genomics. 2016 Aug 11;17:608. doi: 10.1186/s12864-016-2925-6 (PMC4981969; doi:10.1186/s12864-016-2925-6)
Supplement: Additional file 17: Table S17. — List of primers used in quantitative RT-PCR for validation of RNA-Seq analysis. (DOCX 15 kb) [file 12864_2016_2925_MOESM17_ESM.docx]

**Table S17.** List of primers used in quantitative RT-PCR for validation of RNA-Seq analysis

| **Gene ID** | **RAP-DP annotation** | **Forward primer** | **Reverse primer** |
| --- | --- | --- | --- |
| Os12g0443000 | Similar to Cytochrome P450 | CATAACCTGCTATGCCTTGG | TGCGGTAGAAGTCGAGCAG |
| Os01g0860800 | Glycoside hydrolase, family 17 protein | GTACTCGGCGCAGATGTTCT | TGAGGACAGAAGGGGCAATA |
| Os02g0605900 | Similar to Chitinase | GCAAGAACTACTGCGACGAG | GCCCGTAGTTGAAGTTCCAC |
| Os07g0115300 | Similar to Peroxidase2 precursor | TCTCTCAGGCCGACATGATC | CGACCCAAGAATGTGTTGCA |
| Os08g0189600 | Germin-like protein 8-7, Disease resistance | ACCATCTTCCTTCTGCCTCC | GGGCTAGGATCAGAAGCGAT |
| Os02g0787300 | Similar to MAP kinase kinase | TCGAGTTCTACATGGGCAGG | GCGAGTCGGAGTAGCAAATC |
| Os06g0158100 | Similar to WRKY transcription factor 63 | TGCAAGTTTGGAGGCTACAC | GGAATTTGGCACCGAGGATG |
